# Effects of Sleeve Gastrectomy in Neonatally Streptozotocin-Induced Diabetic Rats

**DOI:** 10.1371/journal.pone.0016383

**Published:** 2011-01-21

**Authors:** Yan Wang, Lingling Yan, Zhendong Jin, Xin Xin

**Affiliations:** 1 Department of Pharmacology, Basic Medical School, Tongji Medical College, Wuhan, Hubei, China; 2 Department of Plastic Surgery, Tongji Hospital, Wuhan, Hubei, China; University of Ulster, United Kingdom

## Abstract

**Background:**

Sleeve gastrectomy (SG) has emerged recently as a stand-alone bariatric procedure to treat morbid obesity and enhance glucose homeostasis. The aim of the study was to evaluate its effects in neonatally streptozotocin (STZ)-induced diabetic rats (n-STZ diabetic rats).

**Methodology and Principal Findings:**

To induce diabetes, STZ (90 mg/kg) was administered intraperitoneally to 2-day-old male pups. When 12 weeks old, diabetic rats were randomized into sleeve operation group (SLG, n = 6) and sham operation group (SOG, n = 6). Body weights were monitored weekly, and daily consumption of water and food were followed for eight consecutive weeks postoperatively. Serum glucose levels were measured periodically at the 4th and 8th week after surgery. Insulin, ghrelin, glucose-dependent insulinotropic polypeptide (GIP) and Glucagon-like peptide-1 (GLP-1) levels were assayed at the end of the study. Our data showed that SLG rats exhibited significantly lower body weight gain in addition to reduced food and water intakes postoperatively compared to their sham-operation counterparts. However, resolution of diabetes was not observed in our study. Correspondingly, there were no significant differences between SOG rats and SLG rats in glucose metabolism-associated hormones, including insulin, GIP and GLP-1. In contrast, ghrelin level significantly decreased (P<0.01) in SLG group (58.01±3.75 pg/ml) after SG surgery compared to SOG group (76.36±3.51 pg/ml).

**Conclusions:**

These observations strongly suggest that SG is effective in controlling body weight. However, SG did not achieve resolution or improvement of diabetes in n-STZ diabetic rats.

## Introduction

Sleeve gastrectomy (SG) is one of the restrictive surgical procedures applied for treating morbid obesity consisting of removing the gastric fundus and transforming the stomach into a narrow gastric tube [1]. This surgical procedure was initially performed as the first stage for the biliopancreatic diversion/duodenal switch (BPD/DS) procedure, aiming to reduce operation risks for super-obese or high-risk patients; however, it has been validated as a stand-alone bariatric surgery nowadays [2]–[3]. Moreover, SG has gained increasing popularity with both bariatric surgeons and patients, mainly because of its relative operative simplicity and lower risk profile [4]–[7].

STZ is a chemical substance specifically toxic to pancreatic β cells. When injected into adult rats, STZ can cause type 1 diabetes with severely elevated blood glucose levels. However, when STZ is administered to neonatal rats, the neonates experience acute hyperglycemia within the first few days. The remaining β cells soon regenerate to compensate for the destroyed ones, which makes the rats maintain nearly normoglycaemia during their early lives [8]–[9]. Nevertheless, hyperglycemia re-emerges when the neonatal rats grow into adults, and the rats gradually develop major features described in type 2 diabetes patients (hyperglycemia, polyphagia, polydipsia, polyuria and abnormal glucose tolerance) after adulthood [10]–[12].

Recently, ample evidence demonstrates that SG-induced body weight loss is accompanied by high rates of improvement or resolution of type 2 diabetes mellitus [13]–[14] and other obesity-associated comorbidities such as dyslipidemia [15], steatohepatitis [16] and insulin resistance [17]. Apart from promoting excess weight loss, common explanations for these responses are based on changes in glucose metabolism-associated hormones including ghrelin, insulin, and GLP-1 [13]–[14], [18]. Notably, a recent report demonstrated that while SG led to normalization of glucose levels in exogenous obesity rats, its hypoglycemic effects in Zucker rats or Zucker diabetic fatty rats were slight and transient [18]. Therefore, in order to corroborate the glycemic control effect of SG and provide further evidence for the application of SG in treating type 2 diabetes in the clinical field, the general aim of this study was to verify whether SG could ameliorate hyperglycemia in n-STZ diabetic rats.

We herein evaluated the effects of SG on body weight, intake and glucose metabolism parameters (insulin, GLP-1, GIP, ghrelin and blood glycemia levels) in neonatal STZ-induced diabetes model in rats. Our results showed that SG was effective in restraining body weight gain and reducing food consumption ofn-STZ diabetic rats, with an 8-week follow-up evaluation showing an effect that lasted. Significantly decreased ghrelin level was also observed in our study. However, SG could not attenuate hyperglycemia in n-STZ diabetic rats.

## Materials and Methods

### Animals

The experiments described in this paper were approved by the Ethical Committee on Animal Experimentation of Tongji Medical College, Huazhong University of Science and Technology, China (Approval ID: 00009543). Healthy SD rats from the Center of Experimental Animals (Tongji Medical College, Huazhong University of Science and Technology, China) were maintained for breeding in this research. Male SD pups, aged 48 h±2 h, received STZ (Sigma, USA) injection intraperitoneally at a dose of 90 mg/kg body weight. Normal control pups received vehicle injection of the same volume. STZ with a dose of 90 mg/kg injected intraperitoneally at 2 days of age is usually applied to induce adult-onset type 2 diabetes in neonatal rats [19]. STZ was dissolved in citrate buffer (PH = 4.5) and all procedures were performed on ice and in darkness to avoid the degradation of STZ. All rats were maintained in a controlled environment with a light/dark cycle of 12 h, a temperature of 20±2°C and a humidity of 50±2%. Water and normal rodent chow (60% carbohydrate, 20% protein, 10% vitamin and mineral mix, 5% fat and 5% cellulose) were given to the rats *ad libitum*.

### Methods

#### Prior to intervention

When 12 weeks old, rats were selected for screening by oral glucose test (OGTT). Rats were fasted 12 h, and glucose was administered orally to the rats at a dose of 2.5 g/kg body weight. Serum glucose levels of 0 h, 0.5 h, 1 h, 1.5 h and 2 h were measured. Rats with blood glucose level≥200 mg/dl at 2 h were considered to be diabetic and later included in the research. A total of 12 diabetic rats were randomized into sleeve operation group (SLG, n = 6) and sham operation group (SOG, n = 6). Randomly selected normal control rats made up of the normal control group (NC, n = 6).

#### Day of intervention

Rats were fasted 12 h while water was available *ad libitum* before surgery. Rats were anesthetized with an intraperitoneal injection of 40 mg/kg pentobarbital sodium and placed supinely with the extremities immobilized. Antibiotic prophylactic was applied by injecting penicillin intramuscularly at a dose of 50 mg/kg 30 min prior to the surgery. The abdomen was shaved and midline incision was made with the length being about 3–4 cm in total. Atraumatic hemostatic forceps were placed along the greater curvature from the antrum to the fundus. A scalpel was used to divide the greater curvature along the atraumatic hemostatic forceps removing approximately 70%–80% of the total stomach. The remnant stomach was sterilized with iodine and then closed with 5-0 silk suture by double continuous stitching. Suture line was examined for integrity and an additional stitch was applied when necessary. After the gastric tube was rebuilt, the peritoneal cavity was cleaned with saline and then closed with 3–0 silk suture. For sham operation, the technique consisted of the same procedure described above except for the resection of the stomach. Each animal was given warmed sterile saline subcutaneously after surgery to avoid dehydration, and allowed to recover spontaneously from anesthesia. Rats were returned to individual cages to avoid cannibalism.

#### Postoperative care

Before resuming oral nutrition, Rats were injected subcutaneously with 5 ml sterile saline twice within the first 24 h and 5 ml glucose-saline solution twice a day within the next 48 h. Thereafter, normal chow was offered to the rats in small amounts frequently to prevent early dilation of the stomach for the following 4 consecutive days. Water and chow were given to the rats *ad libitum* after the 8th day.

#### After intervention

Rats were permitted to recover fully within the first 7 days, and daily consumption of water and food of each rat was recorded after the 8th day. Body weights were measured once every week for 7 consecutive weeks postoperatively.

### Biochemical parameters

Blood was drawn from the ophthalmic venous plexus of the rats, with or without 12 h of fasting (measurement of fasting or fed glucose levels), in the morning at the 4th and 8th week after operation. At the end of the study (the 8th week postoperatively), total blood samples drawn in the fasting state, under cold conditions, were pooled for the assay of insulin, GIP, GLP-1 and ghrelin levels. They were treated with an inhibitor of dipeptidyl dipeptidase IV (10 µl/mL blood; Millipore Corporation, USA) immediately after collection, because dipeptidyl dipeptidase IV was a peptide in the blood which degraded GLP-1. Blood samples were centrifuged at 3500 rpm/min for 15 min at 4°C, and the resulting serum samples were stored at −80°C until analysis. Blood glucose was monitored using colorimetric assay kit (BioSino Bio-technology and Science Inc, China). Insulin level was determined by radioimmunoassay (Rat insulin RIA kit, Beijing North Institute of Biological Technology, China). GIP (Millipore Corporation, USA), active ghrelin (Millipore Corporation, USA) and active GLP-1 (Millipore Corporation, USA) levels were measured using commercial ELISA kits. Intra- and inter-assay reproducibility of assay kits data was available in Text S1.

### Statistical Assays

All values were presented as means±SEM. Blood glucose levels were analyzed using one-way ANOVA followed by Turkey's test. All other data were analyzed for statistical significance by unpaired student's test. A P value of less than 0.05 was considered significant.

## Results

All rats survived the surgery and maintained good health condition throughout the experiment.

### Body weight control

**Figure 1:**
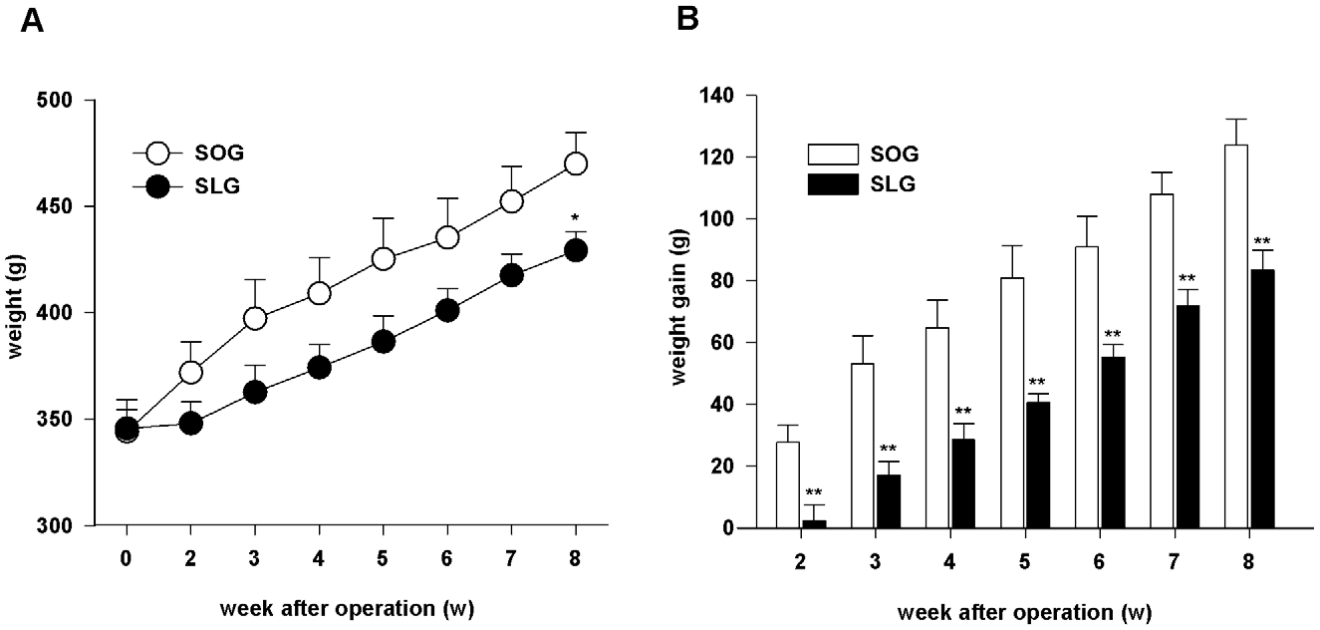
Effect of SG on body weights of rats. (A) Average weight evolution of rats after SG operation. (B) Body weight gain of rats after SG operation. All values are based on the initial body weights of rats. Data are presented as means ±SEM (n = 6 in each group). * P<0.05 and ** P<0.01 *vs*. SOG group.

Evolution of the average weights of SLG rats and SOG rats after surgery was shown in **Figure. 1A**. In the beginning of the study, the two groups had nearly the same average weights (345.67±13.51 g for SLG rats and 344.17±10.44 g for SOG rats). However, SLG rats showed much less weight gain (P<0.01; **Figure. 1B**) postoperatively compared to their sham-operation counterparts. By the end of the study, body weights of the two groups (429.17±8.84 g for SLG rats and 469.83±14.88 g for SOG rats) differed statistically significant (P<0.05; **Figure. 1A**).

### Daily intakes of water and chow

**Figure 2:**
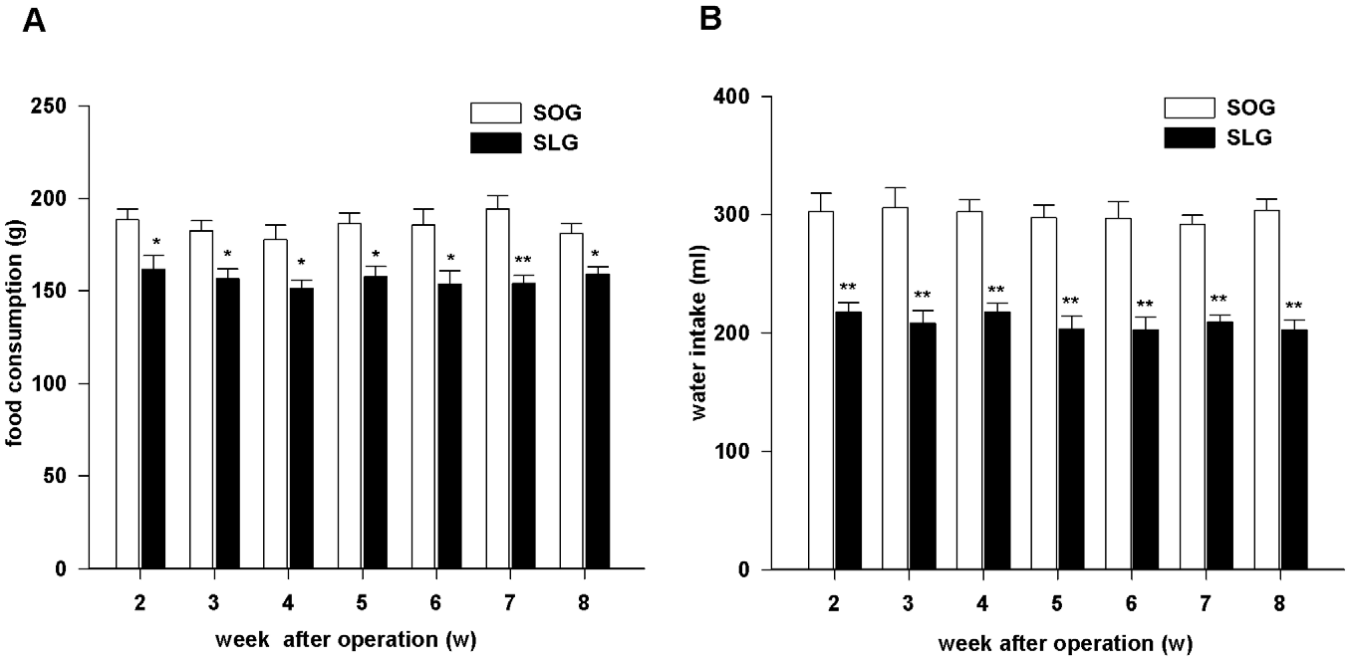
Effect of SG on intakes of rats. (A) Food consumption of rats after SG operation. (B) Water intakes of rats after SG operation. Data are presented as means ±SEM (n = 6 in each group). * P<0.05 and ** P<0.01 *vs*. SOG group.

Daily intakes of water and food were recorded and summarized into the weekly consumption of water and food. There were significant differences in food (P<0.05) and water (P<0.01) consumption between SLG rats and SOG rats throughout the observation period, as shown in **Figure 2**.

### Biochemical parameters

As shown in **Table 1**, administration of STZ to neonatal SD rats resulted in significant hyperglycemia after adulthood. Then-STZ diabetic rats exhibited nearly 2-fold increases in postprandial glucose levels compared to NC rats (P<0.01). Furthermore, there was a progressive deterioration of diabetes in n-STZ diabetic rats. Fasting glucose levels of n-STZ diabetic rats were close to normal (107.57±10.00 mg/dl) at the 4th week after surgery compared with NC rats (90.45±3.60 mg/dl), and increased significantly at the 8th week postoperatively (117.30±4.50 mg/dl for n-STZ diabetic rats and 95.14±2.00 mg/dl for NC rats; P<0.01). Contrary to what we expected, SG did not ameliorate hyperglycemia in n-STZ diabetic rats. Fasting and fed glucose levels of SOG rats and SLG rats, at the 4th and 8th week after SG intervention, were listed in **Table 1**. Moreover, as shown in **Table 2**, there were no significant differences between SOG rats and SLG rats with respect to glucose metabolism-associated hormones, including insulin, GIP and GLP-1. Nevertheless, ghrelin, a gut hormone which was closely involved in appetite regulation, was seen to be significantly decreased (P<0.01) in SLG group (58.01±3.75 pg/ml) compared to SOG group (76.36±3.51 pg/ml), which might partially explain the reduced food intakes and restrained body weight gain of SLG rats after SG intervention.

**Table 1:** Blood glucose levels of rats after SG surgery.

	FAG (mg/dl)		FEG (mg/dl)	
Rat group	4w	8w	4w	8w
NC	90.45±3.60	95.14±2.00	114.59±4.19	119.28±7.21
SOG	107.57±10.00	117.30±4.50**	199.28±10.81**	212.61±11.55**
SLG	105.95±7.43	118.38±5.44**	196.76±11.18**	219.38±15.82**

Data are presented as means ±SEM (n = 6 in each group).

**P<0.01 *vs.* NC group.

FAG: fasting glucose level, FEG: fed glucose level.

**Table 2:** Effect of SG surgery on hormone levels of rats.

	Insulin (µIU/ml)	Ghrelin (pg/ml)	GIP (pg/ml)	GLP-1 (pmol/L)
SOG	29.66±4.50	76.36±3.51	34.36±2.70	4.92±0.43
SLG	25.28±4.64	58.01±3.75**	34.71±3.16	5.74±0.61

Data are presented as means±SEM (n = 6 in each group).

**P<0.01 *vs.* SOG group.

GIP: glucose-dependent insulinotropic polypeptide, GLP-1: Glucagon-like peptide-1.

## Discussion

Sleeve gastrectomy (SG) was initially used as the first stage preceding either duodenal switch or gastric bypass for treating morbid obesity in high-risk patients [20]. However, it has now been validated as an isolated bariatric surgery technique to promote weight loss, which is more rapid and less traumatic [2]–[7], [21]–[23]. In the present work, we studied the effects of SG on body weight, intake and glucose metabolism parameters in n-STZ diabetic rats. We found that SLG rats manifested significantly lower body weight gain along with obvious reduction in food intakes for the 8-week study duration, which provided consistent evidence for the efficacy of SG in controlling body weight [14]–[15], [18], [24].

Although the role of SG in treating morbid obesity is undisputed, its mechanisms remain incompletely understood. Ghrelin is the only gastrointestinal hormone synthesized with a known orexigenic effect, and it is mainly synthesized in the fundus of the stomach [18]. During sleeve gastrectomy, the gastric volume is restricted and the fundus is removed, which significantly decreases the synthesis of ghrelin. This leads to the lowering of appetite and reduction of food intake, which may be the main reason for the sustainable weight loss after SG intervention [24]. Nevertheless, some authors in the literature concluded that the mechanism for SG to promote weight loss was mainly because this “food-limiting” surgery could enhance gastric motility for solids together with the hormone changes [25]. In the current study, significantly decreased ghrelin level was observed in SLG rats postoperatively, which was in agreement with previous observations [14], [26]. Contradictorily, it was found that ghrelin level was relatively low in exogenous obesity rats or genetically determined obesity rats (Zucker rats), and SG did not change [15] or even increased ghrelin level dramatically [18]. Given the physiological role of ghrelin, these heterogeneous findings are understandable, as ghrelin release and gene expression are regulated by the nutrient flux [27] or the nutritional state [28] of the body. Some studies reported that ghrelin level was low in the obese, because it decreased as intake increased [28]. In contrast, ghrelin level was found to be high in conditions of malnutrition or anorexia nervosa, which suggested the possible existence of ghrelin resistance [29]. In the present study, n-STZ diabetic rats developed polyphagia, polydipsia and polyuria after adulthood [12], and collective findings demonstrated that significantly increased ghrelin level in STZ-induced diabetic rats accounted for the diabetic polyphagia [27], [30]–[31]. These have led us to speculate that in the present work, SG-induced body weight loss and food intake reduction are partly, if not totally, due to the ghrelin level change, and furthermore, have reminded us of the “hypoinsulinemia” in type 1 diabetes and “hyperinsulinemia and insulin resistance” in type 2 diabetes, in which both represent the morbid situation deserving appropriate therapies to make them go back to normal.

Recently, substantial evidence has emerged demonstrating the effectiveness of SG in improving or resolving type 2 diabetes [13]–[15], [32]. However, the mechanism for type 2 diabetes resolution after SG is yet to be determined. In fact, not just SG, but other forms of bariatric surgery have been highlighted for their potential to ameliorate hyperglycemia and tackle type 2 diabetes during recent years [33]–[35]. There are two general types of bariatric surgery: restrictive procedures including gastric banding and SG, which physically limit the size of the stomach, and gastrointestinal bypass procedures such as Roux-en-Y gastric bypass (RYGB), which promote the malabsorption of calories [34]. It was assumed initially, that the efficacy of bariatric procedures in treating type 2 diabetes was mainly because of their capacities to promote excess body weight loss [36]. However, some reports later claimed that glycemic control often occurred long before significant weight loss in gastrointestinal bypass procedures such as RYGB [37], which suggested the mechanisms beyond weight loss and calorie restriction. Nevertheless, despite all the promising results obtained up to now, enthusiasm must remain guarded, as most clinical studies were not randomized and thus are suspect with regard to selection and observational bias [34].

In this research, our results showed that SG failed to attenuate hyperglycemia in n-STZ diabetic rats. Supporting this finding, there were no significant changes in the hormones which were closely associated with glucose metabolism, including insulin, GLP-1 and GIP. The reason of the discrepancy may lie in the difference of the model or species. Herein the nature and characteristics of this n-STZ diabetic rat model needs to be considered. STZ is a substance specifically toxic to pancreatic β cells, and the damage STZ causes to β cells leads directly to the dysfunction of β cells which can not be easily reversed. The n-STZ diabetic rats have reduced β-cell mass, decreased pancreatic insulin reserves, and an impaired secretion of insulin to a glucose stimulus [12]. Therefore, it is possible that in these diabetic rats a point of “no return” exists in reversing pancreatic failure. Besides, n-STZ diabetic rats in this study have an uncontrolled disease duration roughly equivalent to 6 human years, which is relatively long [1]. In addition, Pereferrer FS [18] investigated the influence of SG on four experimental models, including non-obesity model, exogenous obesity caused by excessive calorie intake, genetically determined obesity(Zucker rats) and genetically determined obesity and type 2 diabetes mellitus(Zucker diabetic fatty; ZDF rats). Interestingly, it was found that normalization of weight and metabolic parameters were only observed in exogenous obesity model, and effect was slight in Zucker rats or ZDF rats. More broadly, clinical practices have proved that diabetes resolution appears to be more pronounced after gastrointestinal bypass procedures such as RYGB, rather than after the purely restrictive procedures, and in patients with a milder disease or shorter preoperative diabetes duration [34], [38]–[39]. These suggest that the pathogenesis and duration of the disease, the degree of glucose level, and the surgery adopted are important pre-surgical predictors of type 2 diabetes outcome following any type of bariatric surgery.

Taken together, we conclude that SG is effective in controlling body weight. Meanwhile, we speculate that SG is possibly more effective to treat mild diabetes with a shorter duration. When applied to the clinical field, the etiology, pathogenesis and duration of diabetes should all be considered. Moreover, in order to provide better guidelines for the application of this bariatric procedure, further studies are necessary to address putative mechanisms for SG to treat morbid obesity and diabetes.

## Supporting Information

Text S1 Intra- and inter-assay reproducibility of assay kits data.
(DOC)

## References

[1] Patrikakos P, Toutouzas KG, Perrea D, Menenakos E, Pantopoulou A (2009). A surgical rat model of sleeve gastrectomy with staple technique: long-term weight loss results.. Obes Surg.

[2] Fuks D, Verhaeghe P, Brehant O, Sabbagh C, Dumont F (2009). Results of laparoscopic sleeve gastrectomy: a prospective study in 135 patients with morbid obesity.. Surgery.

[3] Sammour T, Hill AG, Singh P, Ranasinghe A, Babor R (2010). Laparoscopic sleeve gastrectomy as a single-stage bariatric procedure.. Obes Surg.

[4] Frezza EE, Reddy S, Gee LL, Wachtel MS (2009). Complications after sleeve gastrectomy for morbid obesity.. Obes Surg.

[5] Tan JT, Kariyawasam S, Wijeratne T, Chandraratna HS (2010). Diagnosis and management of gastric leaks after laparoscopic sleeve gastrectomy for morbid obesity.. Obes Surg.

[6] Akkary E, Duffy A, Bell R (2008). Deciphering the sleeve: technique, indications, efficacy, and safety of sleeve gastrectomy.. Obes Surg.

[7] Goitein D, Goitein O, Feigin A, Zippel D, Papa M (2009). Sleeve gastrectomy: radiologic patterns after surgery.. Surg Endosc.

[8] Li L, Yi Z, Seno M, Kojima I (2004). Activin A and betacellulin: effect on regeneration of pancreatic beta-cells in neonatal streptozotocin-treated rats.. Diabetes.

[9] Thyssen S, Arany E, Hill DJ (2006). Ontogeny of regeneration of beta-cells in the neonatal rat after treatment with streptozotocin.. Endocrinology.

[10] Hemmings SJ, Spafford D (2000). Neonatal STZ model of type II diabetes mellitus in the Fischer 344 rat: characteristics and assessment of the status of the hepatic adrenergic receptors.. Int J Biochem Cell Biol.

[11] Chen D, Wang MW (2005). Development and application of rodent models for type 2 diabetes.. Diabetes Obes Metab.

[12] Takada J, Machado MA, Peres SB, Brito LC, Borges-Silva CN (2007). Neonatal streptozotocin-induced diabetes mellitus: a model of insulin resistance associated with loss of adipose mass.. Metabolism.

[13] Lee WJ, Ser KH, Chong K, Lee YC, Chen SC (2010). Laparoscopic sleeve gastrectomy for diabetes treatment in nonmorbidly obese patients: efficacy and change of insulin secretion.. Surgery.

[14] Li F, Zhang G, Liang J, Ding X, Cheng Z (2009). Sleeve gastrectomy provides a better control of diabetes by decreasing ghrelin in the diabetic Goto-Kakizaki rats.. J Gastrointest Surg.

[15] Lopez PP, Nicholson SE, Burkhardt GE, Johnson RA, Johnson FK (2009). Development of a sleeve gastrectomy weight loss model in obese Zucker rats.. J Surg Res.

[16] Wang Y, Liu J (2009). Sleeve gastrectomy relieves steatohepatitis in high-fat-diet-induced obese rats.. Obes Surg.

[17] Rizzello M, Abbatini F, Casella G, Alessandri G, Fantini A (2010). Early postoperative insulin-resistance changes after sleeve gastrectomy.. Obes Surg.

[18] Pereferrer FS, Gonzàlez MH, Rovira AF, Blasco SB, Rivas AM (2008). Influence of sleeve gastrectomy on several experimental models of obesity: metabolic and hormonal implications.. Obes Surg.

[19] Chakrabarti S, Biswas TK, Seal T, Rokeya B, Ali L (2005). Antidiabetic activity of Caesalpinia bonducella F. in chronic type 2 diabetic model in Long-Evans rats and evaluation of insulin secretagogue property of its fractions on isolated islets.. J Ethnopharmacol.

[20] Saber AA, Elgamal MH, Mcleod MK (2008). Bariatric surgery: the past, present, and future.. Obes Surg.

[21] Sabbagh C, Verhaeghe P, Dhahri A, Brehant O, Fuks D (2010). Two-year results on morbidity, weight loss and quality of life of sleeve gastrectomy as first Procedure, sleeve gastrectomy after failure of gastric banding and gastric banding.. Obes Surg.

[22] Deitel M, Crosby RD, Gagner M (2008). The first international consensus summit for sleeve gastrectomy (SG), New York city, october 25-27, 2007.. Obes Surg.

[23] Bohdjalian A, Langer FB, Shakeri-Leidenmühler S, Gfrerer L, Ludvik B (2010). Sleeve gastrectomy as sole and definitive bariatric procedure: 5-year results for weight loss and ghrelin.. Obes Surg.

[24] de Bona Castelan J, Bettiol J, d'Acampora AJ, Castelan JV, de Souza JC (2007). Sleeve gastrectomy model in Wistar rats.. Obes Surg.

[25] Melissas J, Daskalakis M, Koukouraki S, Askoxylakis I, Metaxari M (2008). Sleeve gastrectomy-a “food limiting” operation.. Obes Surg.

[26] Langer FB, Reza Hoda MA, Bohdjalian A, Felberbauer FX, Zacherl J (2005). Sleeve gastrectomy and gastric banding: effects on plasma ghrelin levels.. Obes Surg.

[27] Gelling RW, Overduin J, Morrison CD, Morton GJ, Frayo RS (2004). Effect of uncontrolled diabetes on plasma ghrelin concentrations and ghrelin-induced feeding.. Endocrinology.

[28] Shiiya T, Nakazato M, Mizuta M, Date Y, Mondal MS (2002). Plasma ghrelin levels in lean and obese humans and the effect of glucose on ghrelin secretion.. J Clin Endocrinol Metab.

[29] Otto B, Cuntz U, Fruehauf E, Wawarta R, Folwaczny C (2001). Weight gain decreases elevated plasma ghrelin concentrations of patients with anorexia nervosa.. Eur J Endocrinol.

[30] Ishii S, Kamegai J, Tamura H, Shimizu T, Sugihara H (2002). Role of ghrelin in streptozotocin-induced diabetic hyperphagia.. Endocrinology.

[31] Tsubone T, Masaki T, Katsuragi I, Tanaka K, Kakuma T (2005). Leptin downregulates ghrelin levels in streptozotocin-induced diabetic mice.. Am J Physiol Regul Integr Comp Physiol.

[32] Rosenthal R, Li X, Samuel S, Martinez P, Zheng C (2009). Effect of sleeve gastrectomy on patients with diabetes mellitus.. Surg Obes Relat Dis.

[33] Abbatini F, Rizzello M, Casella G, Alessandri G, Capoccia D (2010). Long-term effects of laparoscopic sleeve gastrectomy, gastric bypass, and adjustable gastric banding on type 2 diabetes.. Surg Endosc.

[34] Goldfine AB, Shoelson SE, Aguirre V (2009). Expansion and contraction: treating diabetes with bariatric surgery.. Nat Med.

[35] Kindel TL, Yoder SM, Seeley RJ, D'Alessio DA, Tso P (2009). Duodenal-jejunal exclusion improves glucose tolerance in the diabetic, Goto-Kakizaki rat by a GLP-1 receptor-mediated mechanism.. J Gastrointest Surg.

[36] Pories WJ, Swanson MS, MacDonald KG, Long SB, Morris PG (1995). Who would have thought it? An operation proves to be the most effective therapy for adult-onset diabetes mellitus.. Ann Surg.

[37] Herron DM, Tong W (2009). Role of surgery in management of type 2 diabetes.. Mt Sinai J Med.

[38] Buchwald H, Avidor Y, Braunwald E, Jensen MD, Pories W (2004). Bariatric surgery: a systematic review and meta-analysis.. JAMA.

[39] Vidal J, Ibarzabal A, Nicolau J, Vidov M, Delgado S (2007). Short-term effects of sleeve gastrectomy on type 2 diabetes mellitus in severely obese subjects.. Obes Surg.

