# Correction: Effects of Sleeve Gastrectomy in Neonatally Streptozotocin-Induced Diabetic Rats

**DOI:** 10.1371/annotation/06ae95b7-3cf1-44b3-806a-0339ec65092b

**Published:** 2011-02-03

**Authors:** Yan Wang, Lingling Yan, Zhendong Jin, Xin Xin

There is an error in the first affiliation, which affects the first, third, and fourth authors. The correct affiliation is: Department of Pharmacology, Basic Medical School, Tongji Medical College, Huazhong University of Science and Technology, Wuhan, Hubei, China.

