# Supplementary material for: Effects of Sleeve Gastrectomy in Neonatally Streptozotocin-Induced Diabetic Rats
Source: PLoS One. 2011 Jan 21;6(1):e16383. doi: 10.1371/journal.pone.0016383 (PMC3025036; doi:10.1371/journal.pone.0016383)
Supplement: Text S1 — Intra- and inter-assay reproducibility of assay kits data. (DOC) [file pone.0016383.s001.doc]

**Table 1 Within and between assay variation data of active GLP-1 Elisa kit:**

| Sample No. | Mean pM | Within % CV | Between % CV |
| --- | --- | --- | --- |
| 1 | 4 | 8 | 13 |
| 2 | 8 | 7 | 12 |
| 3 | 12 | 6 | 7 |
| 4 | 28 | 7 | 7 |
| 5 | 76 | 9 | < 1 |

Data shown are from four duplicate determinations for within and four duplicate determinations for between.

**Table 2** Within and between assay variation data of active ghrelin Elisa kit

| Sample | Active Ghrelin (pg/mL) Mean | Intra-assay CV (%) | Inter-assay CV (%) |
| --- | --- | --- | --- |
| 1 | 61 | 4.90 | 9.81 |
| 2 | 206 | 1.08 | 2.10 |
| 3 | 1046 | 1.00 | 1.40 |

Intra-assay variations were calculated from results of six duplicate determinations in one assay. Inter-assay variations were calculated from results of six separate assays with duplicate samples in each assay.

**Table 3 Intra-assay variation of GIP Elisa kit:**

| Sample No. | Mean GIP levels (pg/ml) | Intra-Assay % CV |
| --- | --- | --- |
| 1 | 92 | 1.0 |
| 2 | 98 | 2.4 |
| 3 | 173 | 5.9 |
| 4 | 150 | 1.1 |

The mean intra-assay variation was calculated from results of six duplicate determinations in each assay of the indicated samples.

**Table 4** Inter-assay variation of GIP Elisa kit

| Sample No. | Mean GIP levels (pg/ml) | Inter-Assay % CV |
| --- | --- | --- |
| 1 | 80 | 5.9 |
| 2 | 113 | 1.1 |
| 3 | 178 | 4.1 |

The mean inter-assay variation of each sample was calculated from results of four separate assays with duplicate samples in each assay.

**Insulin RIA kit:**

Intra-assay variation <10%; Inter-assay variation <15%

**Glucose colorimetric assay kit:**

Intra-assay variation <2%; Inter-assay variation <3%
